# Changes in Mineral Profile of *Portulaca oleracea* L. Under Foliar Treatment with Sodium Selenate

**DOI:** 10.3390/plants15203150

**Published:** 2026-10-15

**Authors:** Nadezhda Golubkina, Zarema Amagova, Viktor Kharchenko, Lidia Logvinenko, Otilia Cristina Murariu, Gianluca Caruso

**Affiliations:** 1 Federal Scientific Vegetable Center, Moscow 143072, Russia; 2 Chechen Institute of Agriculture, Grozny 366021, Russia; 3 Nikitsky Botanic Garden Russian Academy of Sciences, Yalta 298648, Russia; 4 Department of Food Technologies, ‘Ion Ionescu de la Brad’ Iasi University of Life Sciences, 700490 Iasi, Romania; 5 Department of Agricultural Sciences, University of Naples Federico II, 80055 Portici, Naples, Italy

**Keywords:** purslane, selenium biofortification, toxic elements, roots, leaves, seeds, atomic weight

## Abstract

The mineral composition of edible plants often changes as a result of selenium biofortification, which affects plant species tolerance and adaptability to Se supply, as well as nutritional value. To reveal the peculiarities of macro- and microelements in root–leaf–seed distribution in wild purslane foliar treated with sodium selenate (50 mg L^−1^), 23 chemical elements were determined in control and Se-fortified plants using ICP-MS. Against the backdrop of plant growth stimulation, the concentration of macroelements did not change significantly in roots, increased in leaves due to high Ca and Mg levels, and decreased in seeds (from 16,959 to 11,997 mg kg^−1^ d.w.). A decrease in Fe, Mn, Cu, Zn, and Co was recorded in leaves (1.37–1.69 times) and seeds (1.38–3.07 times), with insignificant changes in mineral profile in roots. Accordingly, the root–leaf Transfer Factor (TF) of Ca and Mg increased while leaf–seed TF decreased. Root–leaf TF of K, Na, and P decreased due to Se supply. No changes were recorded for Si TFs or for Cu leaf–seed TF. On the other hand, the TF increased for B (root–leaf) and Zn (leaf–seed). Changes in potentially toxic element concentrations due to Se supply showed negative correlations with the atomic weight of elements (roots r = −0.886; leaves r = −0.931; *p* < 0.001, and seeds r = −0.867; *p* < 0.005). Similar relationships for several other plant species (*Artemisia annua* L.; *Allium ursinum* L*.*; *Brassica rapa* subsp. *rapa* L.) and a lack of corresponding correlations in other crops (*Allium sativum*, *Allium cepa*, *Brassica oleracea*) indicate the need for further mineral profile investigations in Se-fortified plants.

## 1. Introduction

Intensive climate changes during the last decades increased the frequency of various environmental stresses (drought, flooding, salinity, anomalous temperature fluctuations), which negatively affects the efficiency of crop production and quality, antioxidant status and, in particular, mineral content. To maintain high vitality, plants activate different strategies of adaptation, such as antioxidant defense, osmoregulation, and changes in mineral composition.

The selenium biofortification of plants is known to be an effective approach for improving crop yield and stress tolerance, along with having a significant beneficial effect on human health due to Se status optimization, decreased risks of cardiovascular, oncological, and viral diseases, longevity enhancement, improvement of brain activity and reproduction [1].

The effect of Se application on plant mineral composition reflects a complex relationship with the supplementation method (hydroponics, soil, or foliar supply), the chemical form of the element (organic, inorganic forms, nanoparticles), environmental factors, and interspecies variations [2], providing either beneficial or negative effects. In this respect, ubiquitous utilization of selenate-containing fertilizers in Finland since 1980 provided a valuable optimization of the human Se status [3], accompanied by Zn, Cu, and Cd reduction in certain agricultural crops and unpredictable changes in micro- and other element content [4]. According to the literature reports [5], the intensity of the positive or negative effect of soil selenate supply on the mineral composition of garlic is greatly affected by the stage of plant development, Se dose, elements tested and plant parts. Spyrou et al. [6] demonstrated significant interspecies differences in plant Zn, Mg, Mn, Fe, Cu, and P content changes under SeO_2_ nutrient solution supply in *Taraxacum officinale* and *Mesembryanthemum crystallinum*, with no changes in *Portulaca oleracea* microgreens.

Numerous investigations provide a broad picture of mineral profile improvement due to Se treatments [2,7], along with the absence of significant changes and occasional decrease in certain element content [5,6]. Gui et al. [2] indicated that Se interaction with essential elements may be achieved via changes in the intensity of gene expression of proteins participating in mineral transfer, hormonal regulation, and a competition between the elements. Furthermore, a previous investigation of the mineral profile related to control and Se-treated cabbage revealed that foliar selenate supply significantly changed the relationship between the elements [8].

A comprehensive review on the protective effect of Se in plants against heavy metal accumulation [9] emphasized the existence of a complex protective mechanism via the improvement of the antioxidant defense and photosynthesis, membrane stability, mineral nutrition homeostasis, and a significant decrease in heavy metal absorption by roots and their translocation to the aboveground tissues. Interspecies differences in heavy metal tolerance and the complexity of plant–heavy metal interaction hamper the prediction of the Se beneficial effect value, which may vary significantly depending on genetic and environmental factors [10,11,12].

Among different agricultural crops, *Portulaca oleracea* L. showed high adaptability to environmental stresses, hyper-accumulation of many elements and a remarkable beneficial effect on human health [13], due to the high content of natural antioxidants, K, Mg, P, and especially omega-3 fatty acids [14,15,16]. Biofortification of wild purslane with Se enhanced plant biomass and the content of PUFA in seeds, significantly decreased the oxalic acid levels, entailing its importance in the prophylactics of cardiovascular diseases [17].

Based on what was mentioned above, the present investigation aimed at evaluating the effects of sodium selenate foliar supply on the mineral composition of wild purslane leaves, seeds, and roots.

## 2. Results and Discussion

### 2.1. Macro- and Microelements

#### 2.1.1. Mineral Distribution

Growth optimization is greatly determined by the total amount and plant part accumulation of chemical elements [18]. In this respect, *Portulaca oleracea* draws special attention as a plant with the powerful ability to hyperaccumulate many elements, including potentially toxic ones [19,20]. The detailed mineral profile of purslane (Table 1) revealed specific macro- and microelement distribution between roots, leaves, and seeds in control and Se-fortified plants.

In this respect, seeds of control and Se-fortified purslane showed the highest levels of K, P, Zn, Cu, Mn, and B, indicating the significance of these elements for reproduction [21], and the lowest concentrations of Na, Ca, Si, and Li, compared to leaves and roots. On the other hand, purslane leaves were characterized by the highest concentrations of Mg, Ca, and Li.

**Figure 1:**
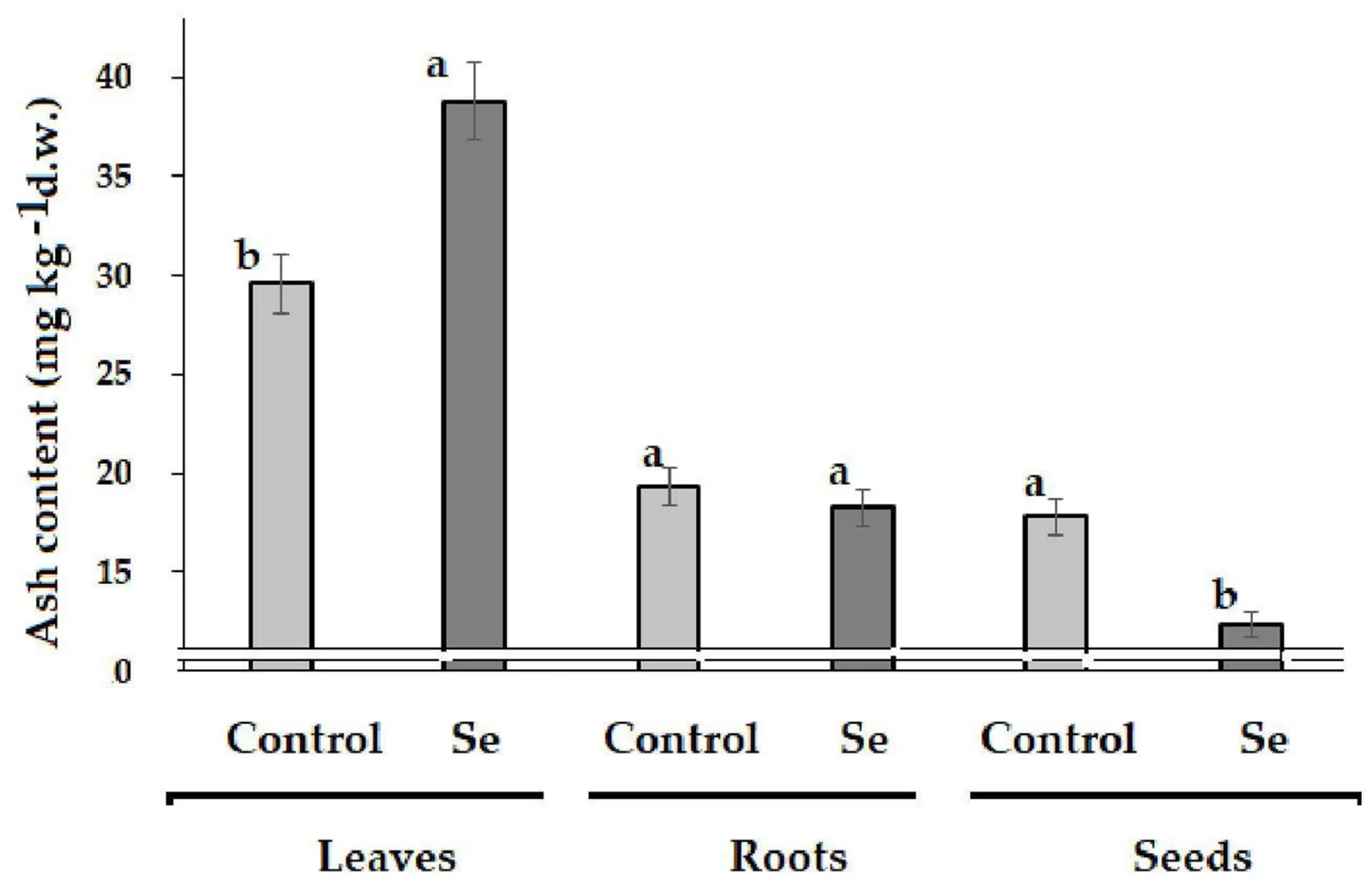
Effect of Se foliar biofortification on ash content in purslane leaves, roots, and seeds. For each plant part, values with the same letters do not differ significantly according to F test (ANOVA) at *p* < 0.05.

The ash content recorded in the different plant parts under selenium supply (Figure 1) indicates the high mineral density of purslane, in accordance with the unusually high purslane ability to assimilate chemical elements [19,20]. Indeed, based on soil Fe/Zn level (242 mg kg^−1^ d.w. and 25.9 mg kg^−1^ d.w., respectively), the coefficient of biological accumulation (CBA) for Fe was close to 1, while Zn CBA values were 1.2 for leaves, 2.56 for roots, and 5.6 for seeds. The ash content decreased from leaves to roots and seeds in control plants, while it significantly increased in leaves and dropped in seeds under Se supply (Figure 1).

**Figure 2:**
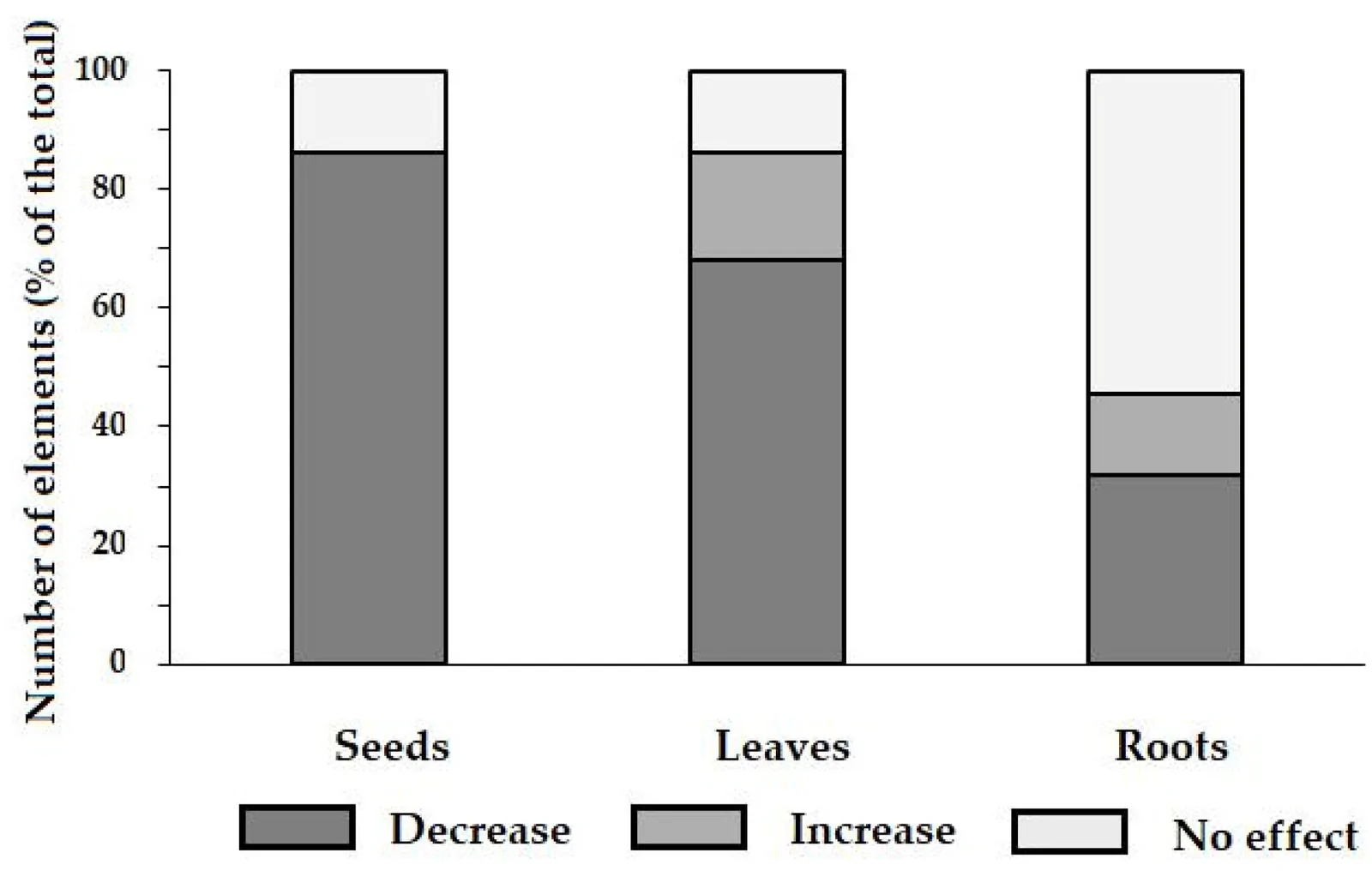
Effect of foliar Se supply on the mineral profile of purslane seeds, leaves and roots.

Furthermore, among the 23 elements tested, a significant decrease in element accumulation was recorded for 19 elements in seeds, 7 in roots and 14 in leaves. On the other hand, there was a significant increase in element levels in four cases in leaves and three in roots, but none in seeds. Interestingly, 12 chemical elements did not change their concentration in roots due to Se supply, while only 3 showed a concentration stability in leaves and seeds (Figure 2).

**Figure 3:**
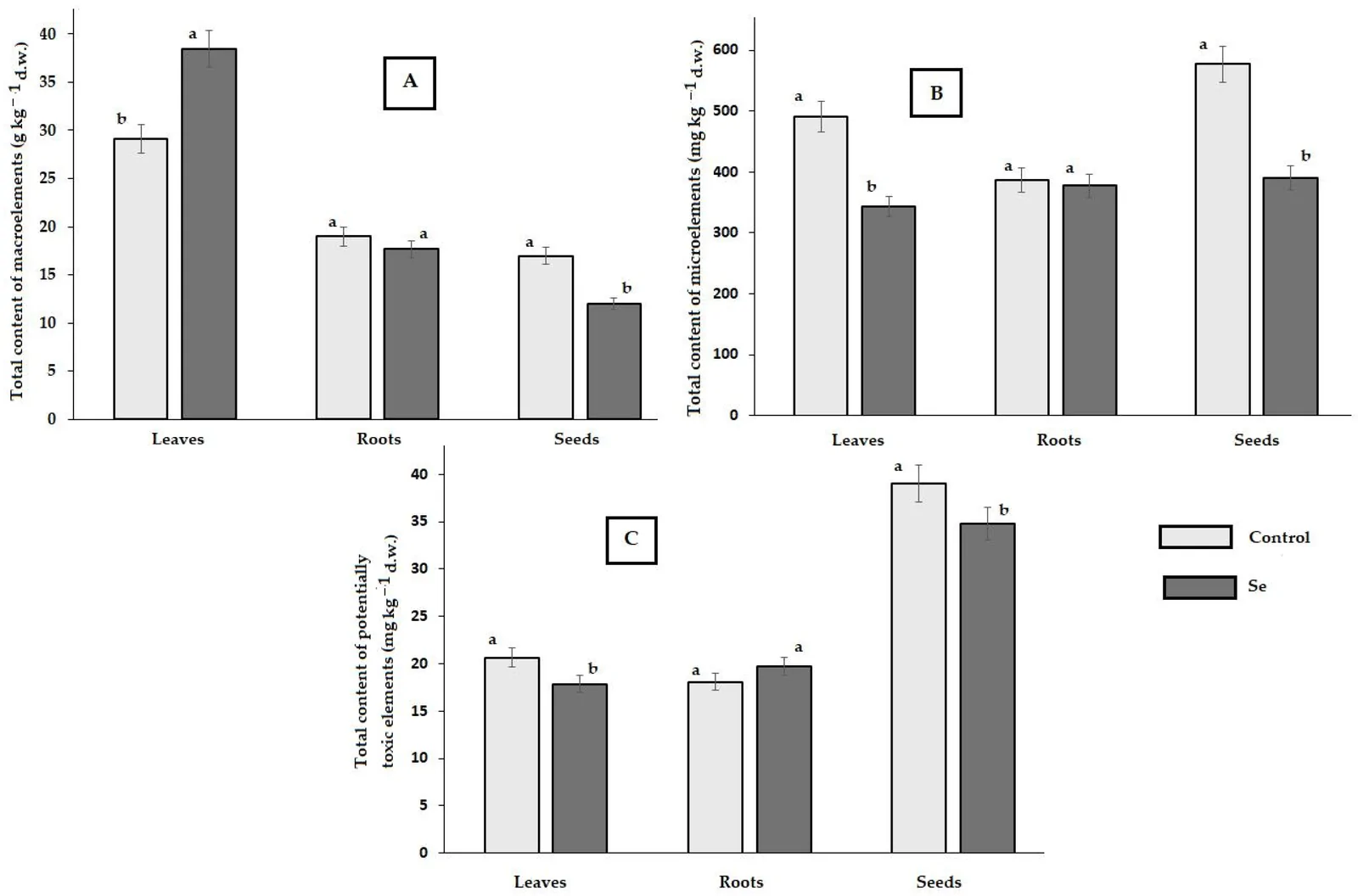
Changes in total macroelements (**A**), microelements (**B**) and potentially toxic elements (**C**) under Se supply. For each plant part, values with the same letters do not differ significantly according to F test (ANOVA) at *p* < 0.05.

A comparison of the total macro-, micro-, and potentially toxic element levels in leaves, roots, and seeds of control and Se-fortified plants (Figure 3A–C) indicated the negative Se effect on these parameters in seeds, while leaves had only a decrease in micro- and potentially toxic elements, but not in macroelements.

The obtained results indicated that the purslane seed mineral profile is the most sensitive to Se supplementation, with the highest dramatic decrease in the total content of macro- (1.41 times), micro- (1.48 times), and potentially toxic elements (2.32 times; excluding Al), compared to leaves and roots. Among potentially toxic elements, only Al content showed no changes in purslane seeds due to plant Se treatment.

The great fluctuations in mineral content in the present investigation, compared to the lack of mineral profile changes recorded in purslane microgreens [6], may be connected with the long vegetation period and multiple Se supplies in our research, which excluded Se toxicosis in plants and even enhanced leaf weight (from 129 g per control plant to 153 g per plant fortified with Se) with a tendency of seed productivity and biomass increase (from 1.79 g per plant to 1.84 g per plant) [17]. This assumption is in accordance with the previous investigations of Perez et al. [5], who reported a significant increase in plant mineral density during the development.

Genetic peculiarities and Se supply method may explain the contradictory results obtained in previous research [22] regarding the increase in Ca, Mg, Mn, Zn, and Cu in maize seedlings under hydroponic supply of selenate and of K and P in blueberry under hydroponics and selenite nutrient supply [23]. The presented results provide additional confirmation of the possible high variability of different plant species’ responses to Se supply. According to Longchamp et al. [22], Ca, Mg, Mn, Zn, and Cu increased in maize seedlings under hydroponic supply with selenate, with no effect on Fe, while a mixture of selenate/selenite decreased the content of these elements. An opposite effect to that recorded in purslane was found in blueberry K and P values and selenite nutrient supply in a hydroponic system [23].

Furthermore, a significant decrease in Zn was recorded in all plant parts: seeds (by 1.48), leaves (1.69), and roots (1.41). Foliar Se supply decreased Zn content in leaves, in accordance with the Finnish investigations of the soil selenate supply effect [4]. A previous investigation also recorded a decrease in Cu and Zn content in oat seeds under selenite foliar supply [24]. Though Se often demonstrates synergy with Zn, several investigations indicate the possibility of a significant Zn decrease under foliar Se supply. Indeed, Nawaz et al. [25] recorded a 54% decrease in Zn in wheat under drought stress and foliar Se treatment. The latter authors explain the aforementioned phenomenon as a reflection of Se influence on ion accumulation in plant cells and possible changes in the permeability coefficient of plasmatic membranes.

Despite the high nutritional value of K for plant survival, purslane prefers to maintain a constant level of the K/Na ratio instead of increasing the K content. Indeed, the K/Na ratio reached 12.9 in leaves, 6.46 in roots, and 48.6 in seeds in control plants and 13.67, 5.52, and 49.4, correspondingly, in plants supplied with Se. Accordingly, at the background of a significant Fe and Mn decrease due to Se supply, the Fe/Mn ratio remained relatively constant: 9.18–8.27 in leaves, 18.48–17.31 in roots, and 3.81–4.00 in seeds. K/Na and Fe/Mn ratios are valuable indicators of ionic homeostasis, stress intensity, and metabolic changes directly connected with osmotic maintenance and photosynthesis in the K/Na case and with redox potential in the other element ratio [26]. The revealed stability of the mentioned ratios in purslane under Se supply indicates plant physiological stability under the chosen conditions and resistance to Se application.

Furthermore, it is worth indicating that habitat significantly affects the chemical element ratio: in the conditions of the present work in Trancaucasia, the macroelement concentrations decreased proportionally both in control and Se-fortified plants according to Ca = Mg > K > P > Na in all plant parts, whereas K prevailed among other macroelements in purslane grown in Malasia [23,27].

#### 2.1.2. The Transfer Factor (TF)

**Figure 4:**
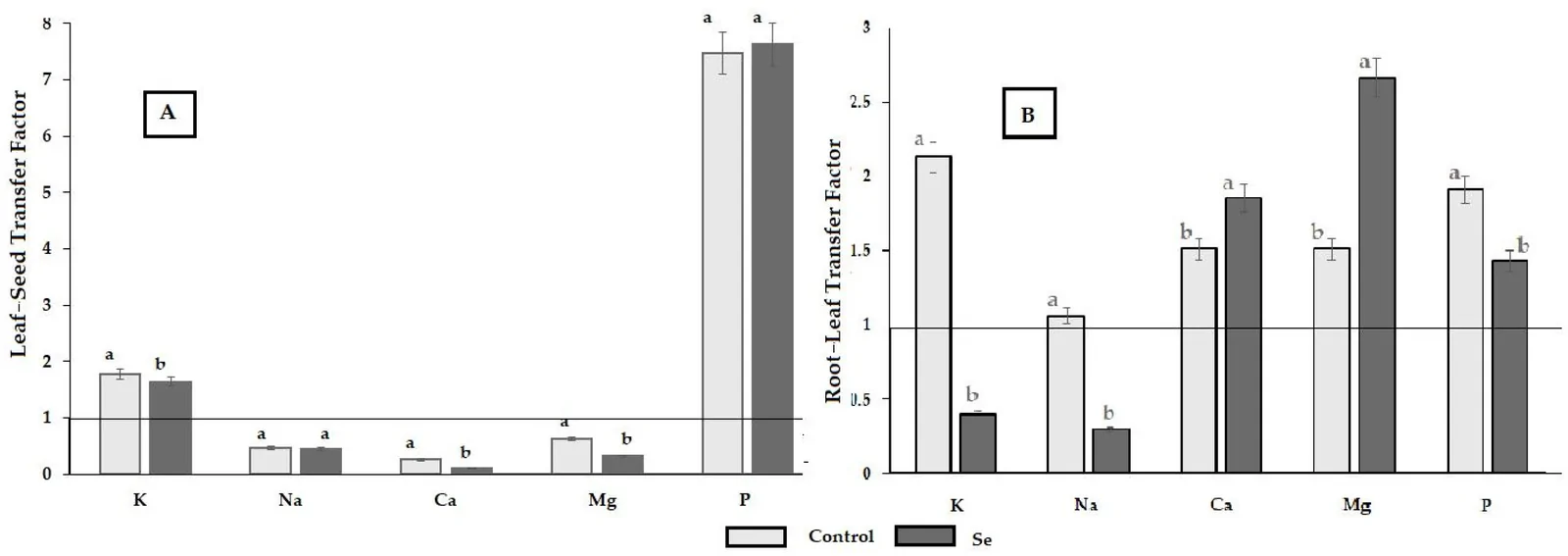
Leaf–seed (**A**) and root–leaf (**B**) TF of macroelements in control and Se-biofortified plants. For each element, values with the same letters do not differ significantly according to F test (ANOVA) at *p* < 0.05.

Root–leaf and leaf–seed Transfer Factors in plants are important indicators of adaptability and survival capacity via changes in chemical element concentration and mineral distribution between plant parts. The observed changes in mineral composition of purslane seeds, leaves, and roots reflected the corresponding significant changes in the Transfer Factors (TFs) due to foliar Se supply. Indeed, the physiological significance of K and P for the progeny was reflected in the highest TF values for these elements in the leaf–seed system and the lowest for Na, Ca, and Mg (Figure 4A). Changes in root–leaf TF were more complex, indicating the increase in this parameter for Ca and Mg, and a decrease for K, Na, and P; for this reason, we assume the participation of Se in the biosynthesis of proteins responsible for mineral transfer [10] (Figure 4B).

**Figure 5:**
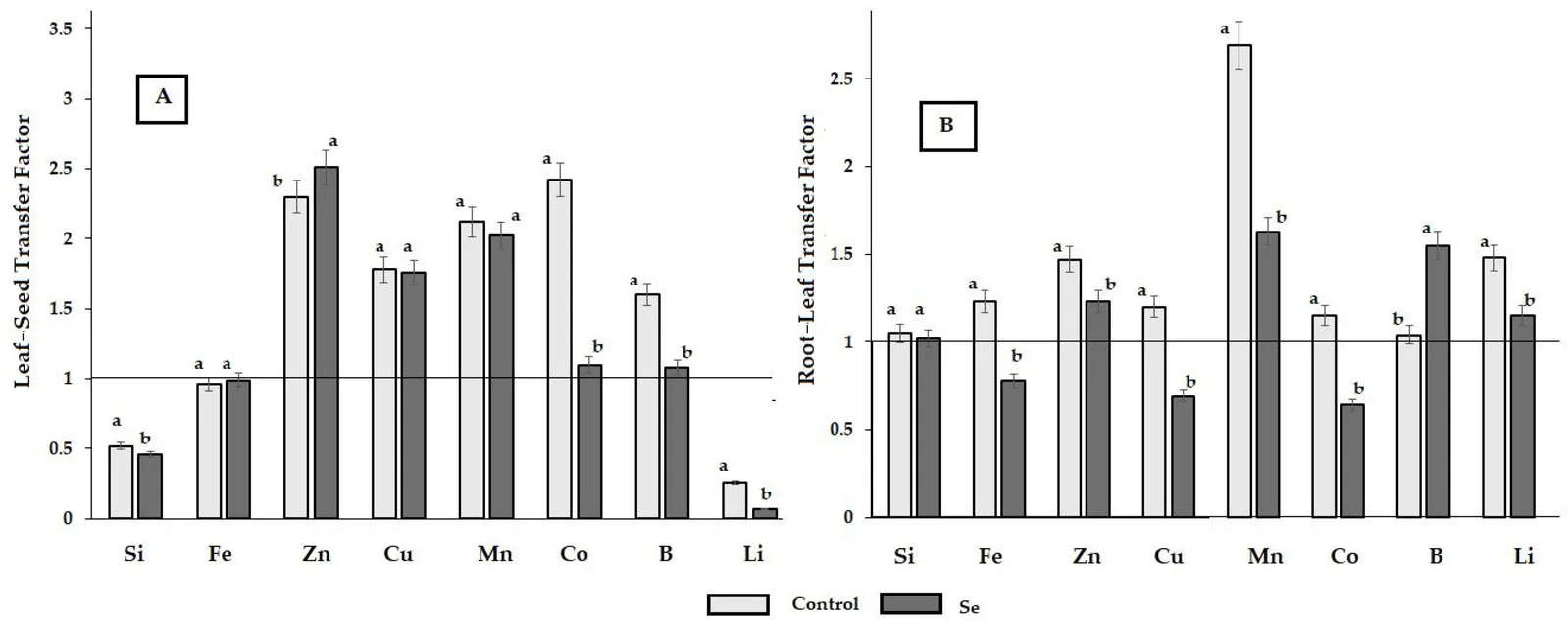
Leaf–seed (**A**) and root–leaf (**B**) TF of microelements in control and Se-fortified plants. For each element, values with the same letters do not differ significantly according to F test (ANOVA) at *p* < 0.05.

Significant changes in the TF were also recorded for microelements, in accordance with the unusual decrease in their concentrations due to Se supply (Figure 5). The presented data indicate low leaf–seed TF for Si and Li (<1) and high TF values (>1) for Zn, Cu, and Mn, with a significant increase in this parameter referring to Zn, as a result of Se supply (Figure 5A). On the other hand, Se reduced the root–leaf TF of Fe, Zn, Cu, Mn, Co, and Li and stimulated the accumulation of B in leaves (Figure 5B), which is in accordance with the importance of B and Zn in reproductive growth and metabolic transport [28], flower development, pollen formation and seed production [29].

The high leaf–seed and root–leaf TF for Mn is in accordance with the important role of Mn in plant physiology [30].

### 2.2. Selenium and Potentially Toxic Elements

Though the division of microelements into essential, beneficial, and toxic categories is rather arbitrary, based on the well-known Shelford’s law of tolerance, we have singled out the following into separate groups of elements with a possible potentially toxic effect: Al, As, Cd, Cr, Hg, Ni, Pb, Sn, and V. The present results indicate the predominance of Cr, Ni, V, and Al accumulations in seeds of control plants, compared to leaves and roots, and close concentrations in leaves of Cd, Pb, and As, which is in accordance with the known heavy metal hyper-accumulation ability of purslane [31]. Foliar supplementation of plants with Se resulted in a dramatic inhibition of most potentially toxic element accumulation, except Al, and Ni in roots and leaves (Table 1). Se supply decreased the concentration of potentially toxic elements in seeds from 1.59 (V), 1.9–2.5 (Sn, Cd, Ni) to 3.8–8.9 (Pb, Hg), and 2.28 (As). The latter effect is especially significant due to the important value of purslane seeds in human nutrition, and their utilization in cardiovascular disease prophylactics based on the extremely high level of omega-3 fatty acids [15]. In this respect, it is worth highlighting that Se biofortification changed As and Cd distribution between seeds, leaves, and roots, leading to the predominant accumulation of these elements in roots but not seeds.

**Figure 6:**
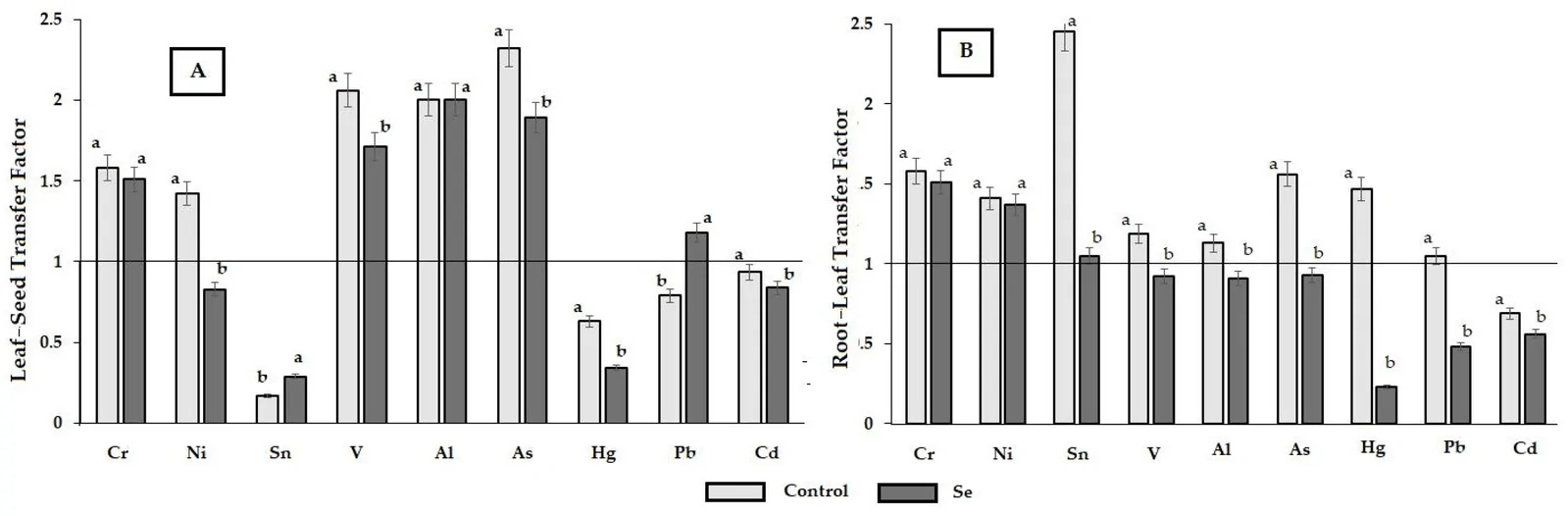
Leaf–seed (**A**) and root–leaf (**B**) TF of potentially toxic elements in control and Se-fortified plants. For each element, values with the same letters do not differ significantly according to F test (ANOVA) at *p* < 0.05.

Changes in the corresponding leaf–seed TF values as a result of Se supply showed the highest decrease for Ni, As, V, and Hg (Figure 6A), indicating the importance of Se-induced seed protection against potentially toxic element accumulation. On the other hand, less pronounced changes in root–leaf TF were recorded, indicating a valuable decrease in the mentioned parameter for most elements, except Cr and Ni (Figure 6B).

Taking into account the relatively negligible seed weight, compared to leaf biomass, despite a significant growth stimulation effect of Se, the phenomenon of lower leaf–seed TF for Se-treated plants becomes even more pronounced while using the values of leaf/seed element content in mg per plant instead of common concentrations (in mg kg^−1^ d.w.), due to the higher conversion factor (K) value for control plants than for Se-purslane:

K for leaf–seed TF of control plants = A × 1.79: (B × 129 × 0.1) = A/B × 0.139;

K for leaf–seed TF for Se-treated plants = A × 1.84: (B × 153 × 0.1) = A/B × 0.120;

where A is the element concentration in seeds in mg kg^−1^ d.w.;

B is the element concentration in leaves in mg kg^−1^ d.w.;
129 is the mean leaf weight of control plants in g [17];
153 is the mean leaf weight of Se-fortified plants in g [17];
1.79 is the ean seed weight of control plants in g [17];
1.84 is the mean seed weight of Se-fortified plants in g [17];
0.1 is the conversion coefficient of leaf f.w. to d.w. [17].

### 2.3. Relationship with the Atomic Weight of Potentially Toxic Elements

**Figure 7:**
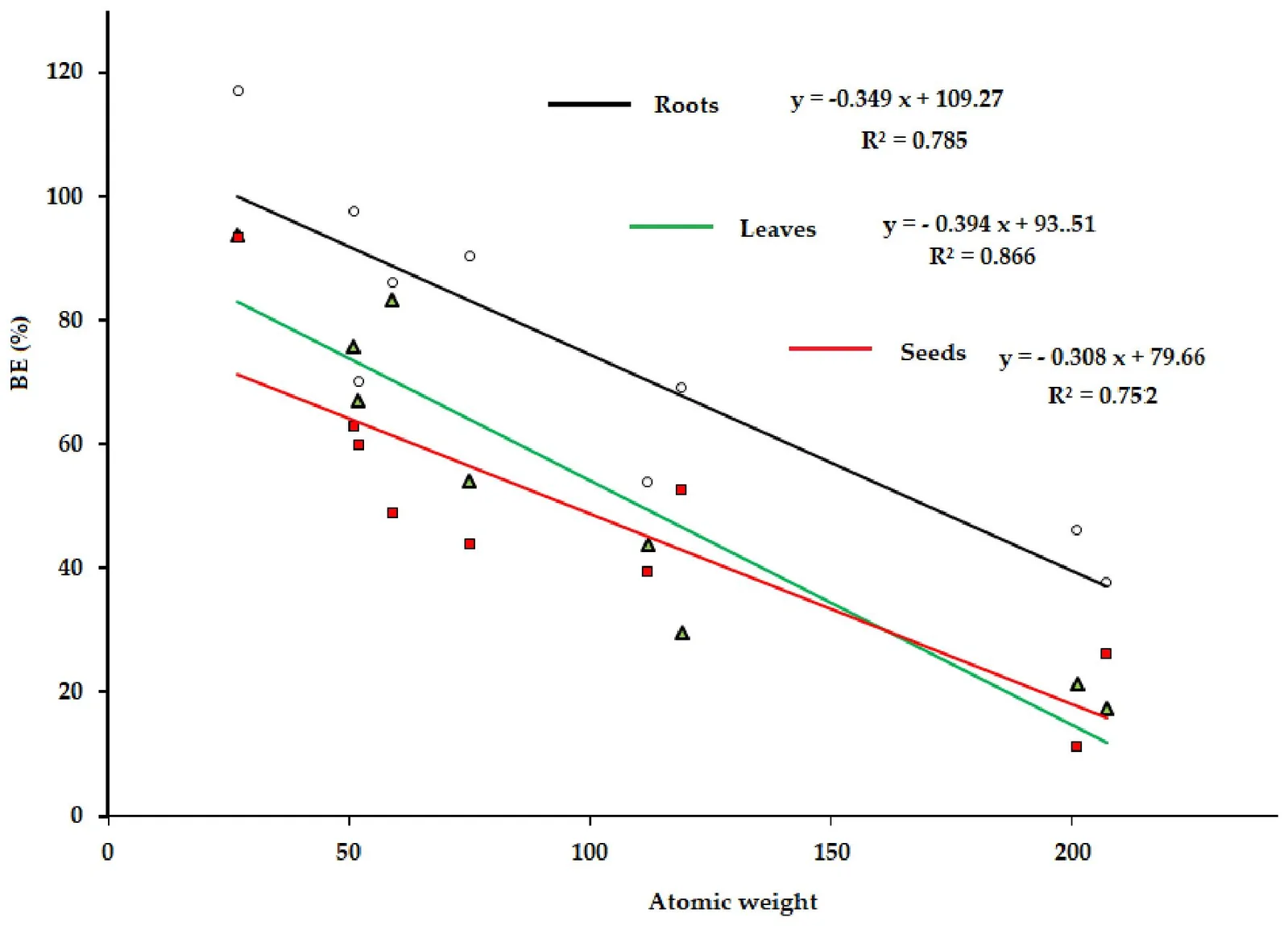
Effect of selenate biofortification of *Portulaca oleracea* with selenium on potentially toxic element accumulation by roots (r = −0.886), *p* < 0.001, leaves (r = −0.931), *p* < 0.001, and seeds (r = −0.867); *p* < 0.005. BE is the potentially toxic element content (%) in plant parts in Se supplied plants, compared to the control.

The classical division of chemical elements into macroelements, microelements, and heavy metals along with As and Al is based on their physiological role and significance in living organism survival [32,33]. The unpredictable variability of mineral profile response to Se treatment in different plant species [2,34] and the significant changes in purslane mineral composition entails the existence of a relationship between plant response to Se treatment and the atomic weight of elements. In this respect, the atomic weight range of the above group of elements varied between macroelements (12–40), microelements (7–65), and potentially toxic elements (27–207). The probability of this inter-relationship between the atomic weight of an element and the change intensity in mineral content due to Se supply should increase along these three groups of elements (macroelements < microelements < toxic elements) according to the values of the atomic weight ranges. Indeed, no relationship between macro- and microelement level changes as a result of Se foliar supplementation to purslane plants was recorded. On the other hand, potentially toxic element data of purslane roots, leaves, and seeds of control and Se-fortified plants revealed significant negative correlations between the parameters, that is, the largest decrease in element concentration due to Se supply corresponds to elements with high atomic weight (Figure 7A–C).

In previous research, the evaluation of the effect of Se foliar supply on the mineral composition of wild plants and agricultural crops revealed significant correlations in *Artemisia annua, Allium ursinum*, and *Brassica rapa* subsp. *Rapa*, similarly to *Portulaca oleracea* (Table 2), and the lack of such a relationship in a collection of cabbages [8]. As far as *Artemisia annua* is concerned, the insignificant Pearson correlation between the above parameters may be connected with the existence of a significantly higher nonparametric Spearman correlation described by the regression equation Y= 17.243 X^−0.694^ with r = −0.865 (*p* < 0.001).

The detected relationships are not sufficient for a definitive phenomenon explanation, but the presented data make it possible to express some important assumptions regarding the factors involved. Indeed, the analysis of possible factors that may affect these relationships revealed that the aforementioned phenomenon may take place both in the case of organic (selenocystine) and inorganic (selenate) Se foliar supply, in conditions of multiple foliar application of the Se-derivatives during vegetation. Furthermore, the related examples (Table 2) indicate the relative soil pH range from 6.2 (with elevated bioavailability of heavy metals) to 7.4 (a decreased heavy metal access). The climate differences associated with the present experiments are deemed not to have significant effects, as the presented relationships (Table 2) were recorded in a continental climate (*Portulaca oleracea*, *Allium ursinum*; Grozny, Chechen republic), temperate continental climate (*Brassica rapa* subsp. *rapa*; Moscow region), and mild subtropical Mediterranean-type climate (*Artemisia annua*; Nikitsky botanic garden).

Notably, the four plants presented in Table 2 (purslane, ramson, Japanese radish, and annual mugwort) showed different tolerances to Se, reflected in significant differences in the biofortification values, varying from 5.75 in *Portulaca oleracea* leaves to 25.6 in *Brassica rapa* subsp. *rapa*. The low ability of purslane to accumulate Se may relate to the significant amount of wax on the leaf surface, which is typical of succulents [37]. At the same time, *Allium ursinum* belongs to secondary accumulators of Se, or Se-indicators [38], characterized by a 14.4 biofortification level, while *A. annua* was moderately affected by Se supply (the biofortification level was equal to 11.3).

The heavy metal tolerance of these plants also differs significantly. Indeed, *P. oleracea* is known to be a hyper-accumulator of heavy metals [39] and, according to the present results, the highest level of heavy metals is typical of plant seeds. *Allium ursinum* exhibits low bioaccumulation capacity for heavy metals, generally acting as a non-accumulator with restricted soil-to-plant metal transfer [40]. *A. annua* demonstrated moderate tolerance to several heavy metals and metalloids, such as Cd, As, and Cr, and is able to accumulate these elements in certain amounts [41]. Among the 11 *Artemisia* species tested in the previous work, *A. annua* demonstrated the highest ability to accumulate Cd [42]. *Brassica rapa* is shown to be a candidate for heavy metal bioremediation due to high tolerance to toxic elements [43].

However, the lack of the corresponding correlations in cabbage biofortified with selenium [8] against the background of similar methods of other plant species’ biofortification may be related, at least partly, to significantly lower levels of potentially toxic elements.

The detected relationships represent an interesting hypothesis related to the mechanism of the Se effect on the potentially toxic element accumulation associated with the element position in the Periodic Table of Chemical Elements. Further investigations are necessary to confirm the above hypothesis based on the effect of different stress factors, genetic peculiarities, Se concentrations, stage of plant development, method of Se supplementation (soil, foliar, hydroponic), and a comparison between Se hyper-accumulators and non-accumulators. Such studies represent a useful contribution to deepening the aspects related to the high variations and controversial results relevant to Se effects on plant mineral composition.

### 2.4. Impact on Human Nutrition

Taking into account the literature data regarding the nutritional value of purslane leaves [44] and seeds [45], the present results provide the first mineral profile peculiarities of wild Transcaucasian purslane biofortified with Se (Table 3).

Compared to 50 g of leaves, 10 g of purslane seeds both in control and Se-treated plants provided significantly higher consumption levels of K, P, Fe, Mn, Cu, Zn, and Se, with the highest levels reaching 30–40% of the RDA level typical for Mn, Fe, and Cu for control plants and 10–25% for Se-fortified plants. Only Ca and Mg showed higher consumption levels with leaves of Se-fortified plants, while the Se concentration in seeds of these plants provides relatively high Se consumption, reaching 8.1% of RDA. These parameters may become valuable for human health maintenance due to the participation of the above elements in antioxidant defense (Se, Mn, Zn, Cu), immunity optimization (Zn, Cu, Se, Fe), energy production (Mn, Fe, Zn, Cu, Se), and bone development (Mn) [1,46,47,48].

**Table 3:** Consumption level of chemical elements with 10 g of wild purslane seeds and 50 g of fresh purslane leaves (% to RDA).

Element	RDA * [49] (mg Day^−1^)	Consumption with 10 g of Seeds		Consumption with 50 g of Fresh Leaves ***	
		Control	Se-Biofortified	Control	Se-Biofortified
K **	2600–3400	0.72–0.95	0.72–0.55	0.20–0.26	0.17–0.22
Ca	1000–1200	3.2–3.9	1.7–2.1	6.3–7.6	7.7–9.3
Mg	310–400	19.3–24.9	14.8–19.1	15.4–19.9	23.0–29.7
P	700	4.0	2.9	0.27	0.19
Fe	8–18	15.6–35.0	10.0–22.5	8.2–18.5	5.0–11.4
Mn	1.8–2.3	31.7–40.6	19.6–25.0	7.6–9.7	4.8–6.1
Cu	0.9	30.0	22.2	8.6	6.2
Se **	0.070	1.0	8.1	0.8	4.4
Zn	8–11	12.8–17.6	8.6–11.9	2.9–4.0	1.7–2.4

* Recommended Dietary Allowance (RDA); ** adequate intake (AI); *** the values were calculated taking into account the dry matter content in purslane leaves (10.1% for control plants and 10.0% for Se-biofortified plants).

However, it is worth mentioning that the relatively low Se biofortification levels for leaves and seeds may be connected with the fact that Se foliar accumulation efficiency is greatly affected by the plant growth phase, i.e., biofortification is more efficient for element uptake and distribution at the vegetative stage than at subsequent stages [50].

Furthermore, other factors affect Se foliar accumulation, such as high temperature, humidity, and light intensity, along with the cuticle thickness, stomatal frequency, intensity of metabolic processes, and interaction with natural antioxidants. From a practical point of view, Se speciation in the resulting product may become the basis for the evaluation of Se bioavailability of purslane treated with sodium selenate and connected with the intensity of inorganic Se conversion to organic derivatives, particularly selenomethionine and selenocysteine [51].

**Figure 8:**
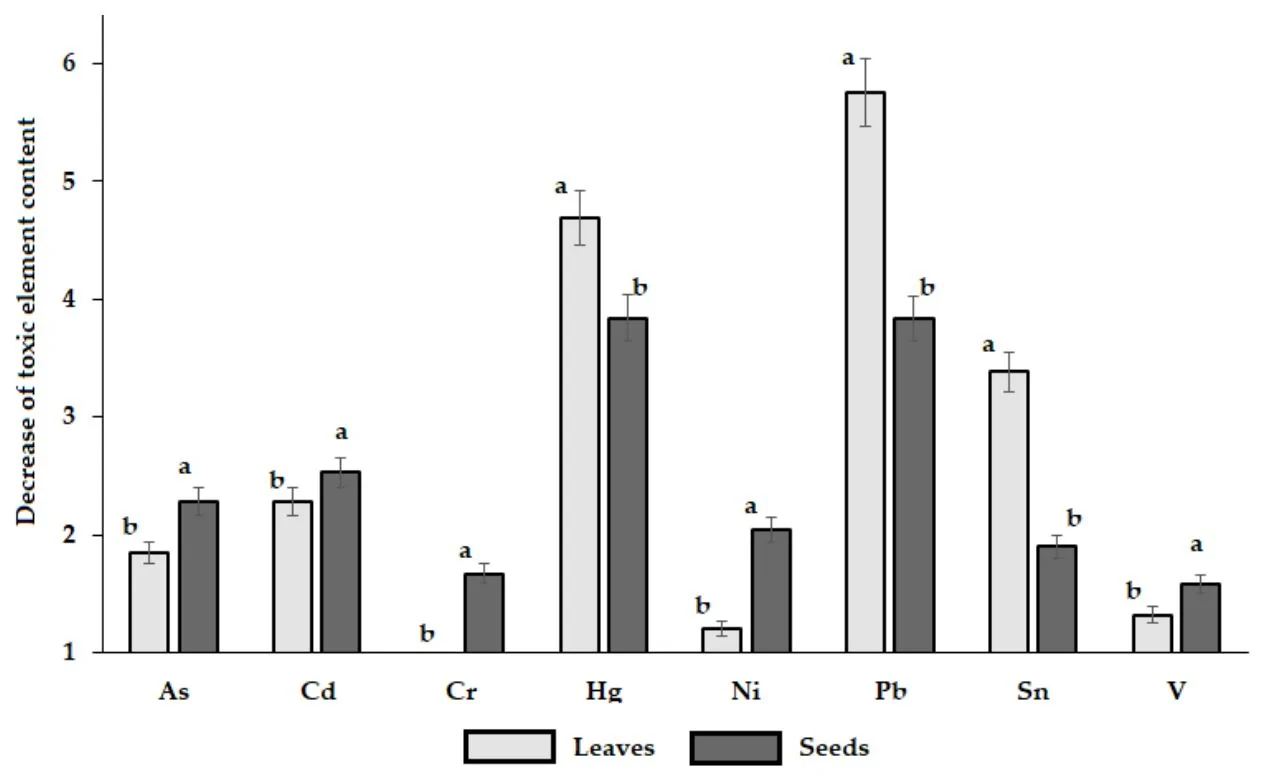
Decrease in toxic element content in leaves and seeds due to foliar Se biofortification of purslane. For each element, values with the same letters do not differ statistically according to F test (ANOVA) at *p* < 0.05.

As far as the potentially toxic elements are concerned, the present results indicate that Se biofortification significantly decreases their consumption both with purslane leaves and seeds, which may become especially important due to purslane’s ability to hyperaccumulate heavy metals [31] against the background of high levels of omega-3 fatty acids and PUFAs [17]. Figure 8 data indicate that Se biofortification is highly valuable in decreasing Pb and Hg concentrations in leaves and seeds, demonstrating the decrease in the beneficial effect value according to the following atomic weight of the elements: Pb ≥ Hg > Cd > As > Ni = V > Cr. This is in accordance with the detected correlations between the intensity of the protection effect of Se against potentially toxic elements and the atomic weight of elements.

Notably, to date, no attempts have been made to evaluate the significance of the relationship between the potentially toxic element’s atomic weight and the beneficial effect of different chemical forms and doses of Se under various methods of Se supplementation, the intensity of environmental stresses, hormonal status, and genetic peculiarities, which restricts the opportunity of the above relationship utilization. However, further investigations are needed to confirm the revealed phenomenon and use the results for developing a direct methodology of plant protection against potentially toxic elements.

## 3. Materials and Methods

### 3.1. Experimental Protocol and Growing Conditions

Research was carried out on wild *Portulaca oleracea* L. grown at the experimental fields of the Chechen Scientific Institute of Agriculture (43◦18′43″ N, 45◦41′20″ E) in 2022 and 2023 in sandy clay loam soil (38% sand, 36% silt, and 26% clay) with pH 7.4 (1:1 soil/H_2_O), 1.3% organic matter, and 200 μg kg^−1^ d.w. Se, 242 mg kg^−1^ d.w. Fe, 145.0 mg kg^−1^ d.w. Mn, 36.2 mg kg^−1^ d.w. Cu, 25.9 mg kg^−1^ d.w. Zn, 24.6 mg kg^−1^ d.w. Pb [17].

The mean values of monthly temperature during the May-September vegetation period were: 16.5-16.6 °C in May; 21.2–21.7 °C in June; 23.7–23.9 °C in July; 23.6–26.1 °C in August; and 17.1–21.0 °C in September. The precipitation values reached 97.5 and 66.0 mm in May; 31.8 and 131.0 in June; 40.3 and 80 mm in July; 9.0 and 12.0 in August; and 31.0 and 84 mm in September.

Both in 2022 and 2023, the experimental protocol was based on the comparison between a foliar selenium treatment and an untreated control, arranging a randomized complete block design with three biological replicates, with the experimental plot covering a 5 m^2^ surface area and containing 42 plants, 20 of which were sampled for analyses.

Foliar treatments of plants with sodium selenate solution (50 mg Na_2_SeO_4_ L^−1^, or 0.26 mM; 0.3 L per m^2^, 60 mg of Na_2_SeO_4_ per m^2^ total) using a Solo hand-held knapsack were performed four times during the 120-day vegetation period, i.e., on 14 July before flowering, 24 July during flowering, and 4 and 14 August during flowering/fruiting. Control plants were treated in a similar way using distilled water. To minimize the number of factors affecting Se accumulation, no fertilizers were applied during the experiment.

At harvest, carried out in the first 10 days of September, both in 2022 and 2023, about 200 g of untreated control and Se-treated purslane plants were collected in each plot. Growth and biochemical determinations were performed separately; the mineral profile was analyzed using homogenized material from the 20 plants sampled. All the analyses were made in triplicate, and the results represent the mean values of 3 biological replicates.

After harvesting, the aboveground part and roots of purslane were washed with distilled water, cut into small pieces and dried at 70 °C to constant weight. The obtained samples along with dried seeds of control and Se-fortified plants were homogenized to a fine powder and used for mineral profile determination.

### 3.2. Plant Elemental Composition

The determination of element concentrations (Al, As, B, Ca, Cd, Co, Cr, Cu, Fe, Hg, K, Li, Mg, Mn, Na, Ni, P, Pb, Sn, Se, Si, V, and Zn) in plants was made in triplicate on the dried homogenized samples of leaves, roots, and seeds, using ICP-MS on a NexION 300D quadruple mass-spectrometer (Perkin Elmer Inc., Shelton, CT, USA), equipped with the seven-port FAST valve and ESI SC DX4 autosampler (Elemental Scientific Inc., Omaha, NE, USA). Microwave digestion of the samples was carried out in sub-boiled HNO_3_, diluted at a ratio of 7:3 with distilled deionized water (Fluka No. 02, 650 Sigma-Aldrich, Co., St. Louis, MO, USA) in the Berghof SW-4 DAP-40 microwave system (Berghof Products + Instruments Gmb H, 72, 800 Eningen, Germany). To eliminate instability during the measurements, an internal standard rhodium 103 Rh was used. Quantitation was performed using external standards (Merck IV, multielement standard solution); the Perkin–Elmer standard solutions for P and V, and all the standard curves, were obtained at five different concentrations. The measurement accuracy and precision were evaluated using the certified reference material NIST 1568b (Rice Flour; Gaithersburg, MD, USA). The limit of detection (LOD), limit of quantification (LOQ), and accuracy, were within the ranges typical for NexION ICP-MS [52]. The appropriate data for Hg, Cd, and Sn are presented in Supplement Table S1.

### 3.3. Ash Content

Ash content in leaves, seeds, and roots of purslane plants was determined heating dry samples from 20 °C to 510 °C to obtain white powder. The related calculation was made using the following equation:

Ash (%) = W_2_: W_1_ × 100,

where W_1_ is the sample weight before the digestion (about 2 g), and W_2_ is the sample weight after the digestion.

### 3.4. Transfer Factor (TF)

The root–leaf and leaf–seed Transfer Factors (TFs) per dry weight for all elements analyzed were determined according to the following:

Root–Leaf TF = C _leaves_: C _roots_

Leaf–Seed TF = C _seeds_: C _leaves_

where C _roots_, C _leaves_, and C _seeds_ are the concentrations of elements in dry roots, leaves, and seeds, respectively.

### 3.5. Biofortification Effect (BE)

To evaluate the relationship between Se supplementation and the atomic weight of elements, the biofortification effect (BE) was calculated according to the following:

BE (%) = (C_Se/_C_control_) × 100,

where C_Se_ is the element concentration in Se-fortified tissue; C_control_ is the element concentration in control tissue.

### 3.6. Statistical Analysis

Both in 2022 and 2023, the data of the 3 biological replicates of both Se treatment and untreated control were statistically processed by the analysis of variance (ANOVA), with reference to the 0.05 probability level, using the SPSS software version 29 according to the following ANOVA scheme: 2 levels of the random experimental factor Year × 2 levels of the fixed experimental factor Se treatment x× 3 Replicates (12 observations overall). As no significant differences between the two research years and no significant interactions between the research year and Se treatment arose, the presented results are the average values (3 replicates) of the two research years.

## 4. Conclusions

The analysis of the Se supplementation effect on the wild purslane mineral profile and element distribution among the plant parts indicated an intensive decrease in almost all element content in seeds, relative stability in roots, and significant changes in macro- and microelement concentrations along with heavy metal decrease in leaves. The identification of high leaf–seed TF > 1 (K, P and Zn, Cu, Mn, Co, B, along with Cr, V, Al, and As) and low leaf–seed TF < 1 (Sn, Hg, Si, Li, Na, Ca, Mg) both in control and Se-treated plants reflects peculiarities of purslane physiology, the hierarchical importance of elements, and high mineral density of seeds. The significant correlations between toxic element content changes due to Se supply and their atomic weight was reported for purslane roots, leaves, and seeds along with the corresponding correlations for *Artemisia annua*, *Allium ursinum*, and *Brassica rapa*. Further investigations are needed to confirm the above hypothesis of the correlations between the intensity of mineral changes due to Se supply and the atomic weight of an element.

## Figures and Tables

**Table 1:** Mineral profile (mg kg^−1^ d.w.) of leaves, roots, and seeds in purslane plants fortified and non-fortified with selenium.

Element	Leaves		Roots		Seeds	
	Control	Se	Control	Se	Control	Se
K	1378.62 a	1147.79 a	647.68 a	462.55 b	2460.44 a	1882.13 b
Na	106.635 a	83.937 b	100.216 a	83.862 a	50.609 a	38.119 b
Ca	15,016.3 b	18,558.5 a	9931.8 a	10,040.8 a	3890.2 a	2098.6 b
Mg	12,220.9 b	18,402.7 a	8091.5 a	6905.5 a	7725.1 a	5935.9 b
P	379.035 a	267.694 b	198.049 a	187.344 a	2832.350 a	2042.580 b
B	10.735 b	15.857 a	10.278 a	10.248 a	17.223 a	17.143 a
Co	0.370 a	0.266 b	0.322 b	0.418 a	0.897 a	0.292 b
Cu	15.265 a	11.116 b	12.754 b	16.124 a	27.186 a	19.562 b
Fe	292.488 a	182.352 b	238.191 a	234.123 a	280.100 a	180.279 b
Li	1.637 a	1.457 a	1.105 a	1.265 a	0.421 a	0.0958 b
Mn	34.639 a	22.038 b	12.889 a	13.534 a	73.461 a	44.574 b
Se	0.108 b	0.621 a	0.061 b	0.593 a	0.070 b	0.570 a
Si	71.052 a	71.317 a	67.441 a	70.048 a	36.679 a	32.647 a
Zn	64.210 a	37.985 b	43.571 a	30.825 b	140.763 a	95.186 b
Al	17.892 a	16.762 a	15.772 a	18.468 a	35.852 a	33.443 a
As	0.211 a	0.114 b	0.135 a	0.122 a	0.490 a	0.215 b
Cd	0.0866 a	0.0379 b	0.1250 a	0.0675 b	0.0810 a	0.0320 b
Cr	0.160 a	0.107 b	0.101 a	0.071 b	0.447 a	0.268 b
Hg	0.0169 a	0.0036 c	0.0115 a	0.0053 b	0.0107 a	0.0012 b
Ni	0.390 a	0.325 a	0.276 a	0.238 a	0.553 a	0.271 b
Pb	1.444 a	0.251 b	1.376 a	0.520 b	1.133 a	0.296 b
Sn	0.2390 a	0.0708 b	0.0974 a	0.0673 b	0.0397 a	0.0209 b
V	0.198 a	0.150 b	0.167 a	0.163 a	0.408 a	0.257 b

For each plant part and element, values with the same letters do not differ significantly according to F test (ANOVA) at *p* < 0.05.

**Table 2:** Relationship between changes in mineral content and atomic weight.

Plant Species	Se Form *** Date of Treatment	Plant Part	r	Regression Equation	Soil pH	Se Biofortification
*Brassica rapa* subsp. *rapa*	SeCys_2_ 20,27 July	Leaves	−0.864 b	Y= −0.002 X + 0.967	6.7	25.5
		Roots	−0.827 c	Y= −0.002 X + 0.922		25.6
Artemisia annua *	Selenate 15 June, 10 August	Leaves	−0.410 d	Y= −0.006 X + 1.499	6.2	11.3
Allium ursinum **	Selenate April 3 times with 10 day interval	Leaves	−0.848 c	Y= −0.004 X + 1.488	7.2	14.4
*Portulaca oleracea*	Selenate 14 and 24 July, 4 and14 August	Roots	−0.886 a	Y= −0.349 X + 109.27	7.4	6.44
		Leaves	−0.931 a	Y= −0.394 X + 93.51		5.75
		Seeds	−0.867 b	Y= −0.308 X + 79.66		6.55

* [35]; ** [36]; *** Se concentration 0.265 mM (a) *p* < 0.001; (b) *p* < 0.005; (c) *p* < 0.01; (d) *p* > 0.5.

## Data Availability

The original contributions presented in this study are included in the article/supplementary material. Further inquiries can be directed to the corresponding authors.

## Supplementary Materials

The following supporting information can be downloaded at: https://www.mdpi.com/article/10.3390/plants15203150/s1, Table S1: LOD, LOQ and recovery values for Cd, Sn, and Hg in NIST 1568b reference material (rice flour).
