# Supplementary material for: Changes in Mineral Profile of Portulaca oleracea L. Under Foliar Treatment with Sodium Selenate
Source: Plants (Basel). 2026 Oct 15;15(20):3150. doi: 10.3390/plants15203150 (PMC13708727; doi:10.3390/plants15203150)
Supplement: Supplementary file 1 [file plants-15-03150-s001.zip › Supplementary-Table S1.pdf]

Table S1. LOD, LOQ and recovery values for Cd, Sn, and Hg in NIST 1568b reference material (rice flour)

Parameter	Cd	Hg	Sn
Certificate value (mg kg^{-1} d.w.)	0.0254	0.0005	0.0030
Determined concentration (mg kg^{-1} d.w.)	0.0236±0.0013	0.0006±0.0001	0.0034±0.0004
Recovery (%)	93.0	120.0	113.3
LOD (μg kg^{-1} d.w.)	0.0.015	0.06	0.12
LOQ (μg kg^{-1} d.w.)	0.07	0.25	
